# Current Advances in Immunotherapy for Glioblastoma

**DOI:** 10.1007/s11912-020-01007-5

**Published:** 2021-01-26

**Authors:** Abigail L. Mende, Jessica D. Schulte, Hideho Okada, Jennifer L. Clarke

**Affiliations:** 1 Department of Neurological Surgery, University of California, Diller Family Cancer Research Building HD 472, Box 520, 1450 3rd Street San Francisco, Helen, CA 94158 USA; 2 Department of Neurology, University of California, San Francisco, CA USA; 3 The Parker Institute for Cancer Immunotherapy, Diller Family Cancer Research Building HD 472, Box 520, 1450 3rd Street San Francisco, Helen, CA 94158 USA; 4 Cancer Immunotherapy Program, University of California, San Francisco, CA USA; 5 Department of Clinical Neurology and Neurological Surgery, University of California San Francisco, Box 0372, 400 Parnassus Avenue, A895F, San Francisco, CA 94143-0372 USA

**Keywords:** Glioblastoma, Immunotherapy, Cancer vaccine, CAR-T, Oncolytic virus, Checkpoint inhibitor

## Abstract

**Purpose of Review:**

This review seeks to inform oncology clinicians and researchers about the development of novel immunotherapies for the treatment of glioblastoma. An enumeration of ongoing and recently completed clinical trials will be discussed with special attention given to current technologies implemented to overcome central nervous system–specific challenges including barriers to the peripheral immune system, impaired antigen presentation, and T cell dysfunction.

**Recent Findings:**

The success of immunotherapy in other solid cancers has served as a catalyst to explore its application in glioblastoma, which has limited response to other treatments. Recent developments include multi-antigen vaccines that seek to overcome the heterogeneity of glioblastoma, as well as immune checkpoint inhibitors, which could amplify the adaptive immune response and may have promise in combinatorial approaches. Additionally, oncolytic and retroviruses have opened the door to a plethora of combinatorial approaches aiming to leverage their immunogenicity and/or ability to carry therapeutic transgenes.

**Summary:**

Treatment of glioblastoma remains a serious challenge both with regard to immune-based as well as other therapeutic strategies. The disease has proven to be highly resistant to treatment due to a combination of tumor heterogeneity, adaptive expansion of resistant cellular subclones, evasion of immune surveillance, and manipulation of various signaling pathways involved in tumor progression and immune response. Immunotherapeutics that are efficacious in other cancer types have unfortunately not enjoyed the same success in glioblastoma, illustrating the challenging and complex nature of this disease and demonstrating the need for development of multimodal treatment regimens utilizing the synergistic qualities of immune-mediated therapies.

## Introduction

Glioblastoma (GBM) is the most common malignant primary brain tumor, affecting approximately 3 out of 100,000 individuals in the USA [1]. Despite an aggressive standard of care regimen of maximal surgical resection followed by combination radiation and alkylating chemotherapy, prognosis remains poor with a 100% recurrence rate and a median overall survival (mOS) of approximately 20 months [2]. Interest in immunotherapy as an alternative approach has rapidly amplified in recent years, following the success of immune checkpoint inhibitors (ICIs) as well as oncolytic viral (OV) therapies in melanoma and other cancers [3–5]. In GBM, however, immune-based therapies have not enjoyed the same success, as demonstrated by the disappointing outcomes of recent randomized, advanced phase clinical trials [6, 7, 8•]. This review will discuss the current status of immunotherapy for GBM, covering inherent neuroanatomical and immunosuppressive challenges in GBM, as well as ongoing research including cell-based therapy, ICIs, vaccines, and viral therapy.

## Neuroanatomical Barriers of Immunotherapy

The central nervous system (CNS) has long been believed to be an immune-privileged environment. However, recent live-imaging studies revealed a CNS lymphatic system with activated T cells crossing the blood-brain barrier (BBB), thus giving rise to new questions about the interaction of the CNS with the peripheral immune system [9–11].

**Fig. 1:**
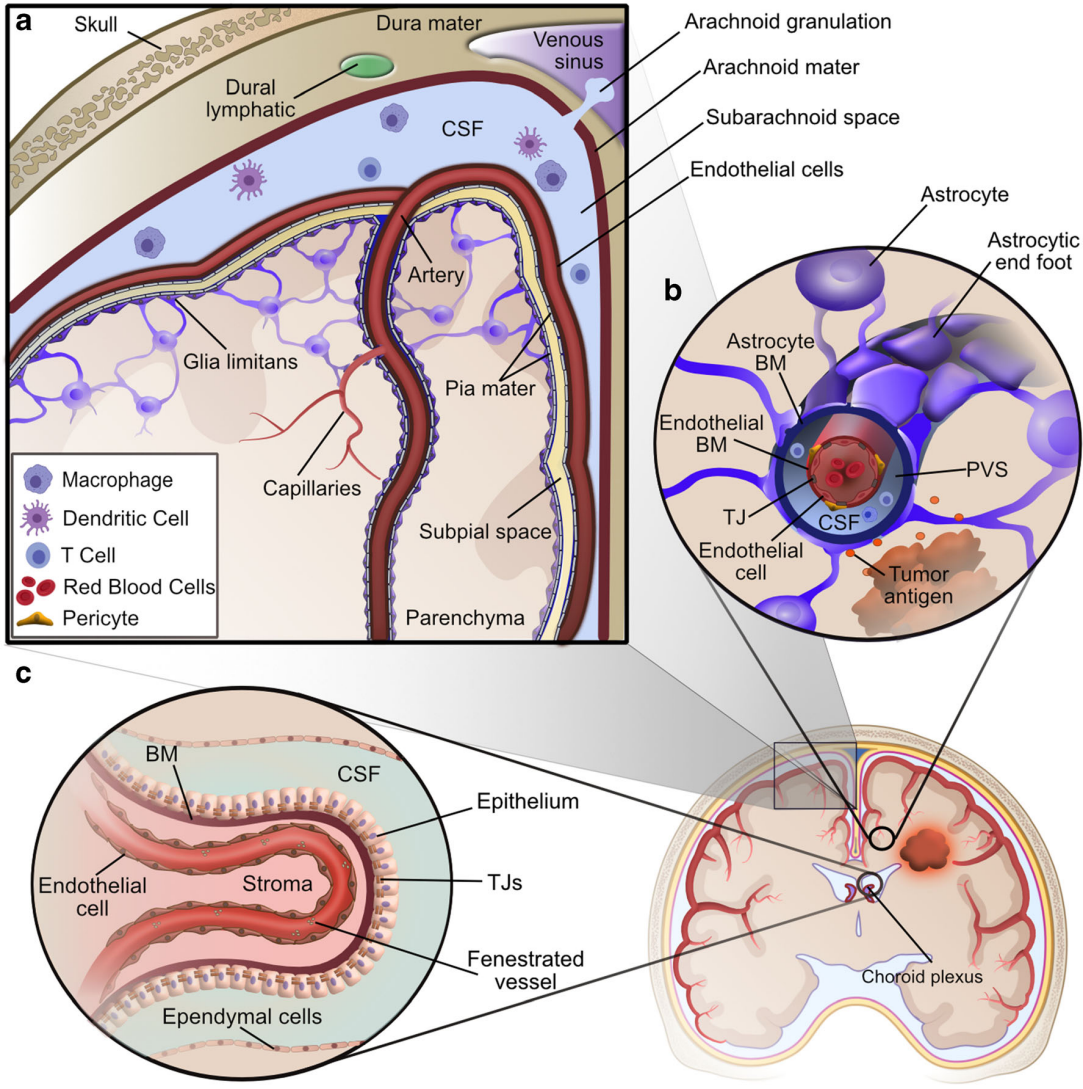
Central nervous system barriers to immunological communication. **a** The blood leptomeningeal barrier at the surface of the brain is composed of the glia limitans and pia mater covering the arteries, while veins exiting the parenchyma are protected only by the glia limitans. Additionally, the subarachnoid space is partitioned from the dural lymphatic system by the arachnoid mater, with the exception of arachnoid granulations allowing regulated drainage into the venous sinus. **b** The blood brain barrier deep in the brain parenchyma is composed of endothelial cells tightly linked together by tight junctions (TJ) and is further enforced by an endothelial basement membrane (BM). In the case of the post-capillary venule shown here, the endothelial BM is separated from the lining of glia limitans formed by astrocytic foot processes and the astrocytic BM, leaving a small perivascular space (PVS) filled with cerebral spinal fluid (CSF), in which immune cells can circulate and potentially interact with CNS antigens. **c** The choroid plexus tightly regulates parenchymal solute exchange with the CSF and inner stroma using a layer of epithelial cells, the epithelium, linked by tight junctions as well as a layer of ependymal cells (*Adapted from Engelhardt, B* et al. *2019)* Created with BioRender.com and Affinity Designer software

While evidence of CNS interaction with the immune system has been noted, three primary barriers deter entry of immune cells into the CNS: the blood-leptomeningeal barrier at the superficial surface, the BBB in the deep parenchyma-penetrating capillaries, and the choroid plexus epithelium (Fig. 1) [12••]. On the superficial surface of the brain, the pial meningeal layer coats arteries as they penetrate the brain surface, but does not coat veins exiting the parenchyma. A second layer of protection is provided by the glia limitans, composed of astrocyte foot processes, which re-enforces the basement membrane of arteries and veins as well as the superficial parenchyma itself. Low-molecular-weight molecules and fluid can pass through the glia limitans, but there is only limited exchange of T cells at this blood-leptomeningeal barrier [13–16]. Deeper in the brain, the BBB restricts the flow of systemic immune cells into the parenchyma. It is formed by specialized endothelial cells with tight junctions, the endothelial basement membrane enforced by pericytes, and the glia limitans. In the post-capillary venules, the endothelial basement membrane is separated from the glia limitans, creating a perivascular space in which immune cells may circulate for potential access to CNS antigens and entry into the CNS. Finally, the choroid plexus epithelium regulates passage of immune cells and solutes into the cerebrospinal fluid (CSF) through specialized tight junctions and efflux pumps [12••].

## Impaired Antigen Presentation Conceals Immune System Awareness of GBM

Antigen-presenting cells (APCs) have four routes from the CNS to reach lymph nodes where the immune response can be initiated. To reach deep cervical lymph nodes, CSF can either drain through the arachnoid villi to central venous sinuses or drain from the subarachnoid space to the lymphatics through the cribriform plate [10, 17]. CSF can also drain through dural lymphatic vessels in the skull base and through the perineurium of cranial and spinal nerve roots [9, 18]. These subarachnoid and dural lymphatic pathways allow some immune cell trafficking near the brain surface. By comparison, surveillance immune cells have limited access to the CNS parenchyma except in the post-capillary venules as described above. Interstitial fluid (ISF) flows though the basement membrane of arteries and capillaries, and this narrow pathway limits trafficking of large molecules as well as immune cells [19]. CSF and ISF exchange to a small degree; studies show low-molecular-weight tracers injected into the CSF enter the ISF through aquaporin-4 channels in the astrocyte end feet of the glia limitans [14, 20]. However, age-related protein deposition into blood vessel walls can limit ISF exchange over time [19, 21]. These forces collectively result in a more immune-privileged environment in the deep parenchyma where GBM often arises. Although the BBB may be partially disrupted in higher grade gliomas, there often remains a significant population of diffusely infiltrative tumor cells deep in the parenchyma that have an intact BBB and therefore may not be as readily accessed by circulating immune cells [22, 23].

The issue of restricted immune cell access is further compounded by the limited habitation of B and T cells in normal parenchyma, although CD4+ and CD8+ T cells can become activated by specific antigen either locally or systemically and then traverse the BBB, even in the absence of neuroinflammation [24, 25]. Entry of activated T cells into the brain parenchyma can occur through the dynamic interaction of adhesion and signaling molecules expressed by immune cells and endothelial cells lining post-capillary venules in parenchymal perivascular spaces [26•]*.*

## Tumor Heterogeneity Limits Degree and Durability of Response to Therapy

Tumor heterogeneity in GBM seriously undermines robust and durable responses to therapeutic interventions, driving poor survival outcomes. In addition to significant genetic diversity between different GBMs, there is substantial subclonal diversity within individual tumors, as defined by genetic and epigenetic profiling [27–31]. Furthermore, single-cell transcriptional profiling has demonstrated that individual GBM cells have temporal variation in gene expression regulating cellular function and the balance between stem-like and differentiation states [32]. In addition, exposure to chemotherapy and radiation, as well as microenvironmental differences in oxygen and other nutrients, can selectively pressure the expansion of certain subclones, or induce hypermutation [33].

Common genetic mutations in adult GBM frequently targeted by immunotherapy include the epidermal growth factor receptor variant III (EGFRvIII) mutation and the isocitrate dehydrogenase (IDH1) R132H mutation, while targets in pediatric gliomas include a conserved missense mutation in histone H3 from lysine (K) to methionine (M) at position 27 (H3 K27M mutation) [34–36]. While most tumor-specific antigens are expressed heterogeneously within tumor, the H3 K27M mutation is homogeneously distributed throughout the entire tumor [37, 38], suggesting that this may be a truncal mutation and less likely to lead to antigen loss–mediated escape of the tumor when targeted therapeutically.

## Immunosuppression in Glioblastoma

Glioblastoma can impair both local CNS and systemic immune system functions, by hijacking major immunogenic signaling pathways and altering cellular immunity inside and outside the brain. In addition, there are immunosuppressive side effects of chemotherapy and radiation.

### The Impact of Glioblastoma on Immune Cell Composition and Functionality

GBM alters physical and functional aspects of the brain immune system through production of immunosuppressive factors and modulation of cell surface receptors and immune cell subsets [39]. GBM induces systemic sequestration of naïve T cells in the bone marrow due to downregulation of sphingosine 1-phosphate receptor, contributing to low levels of effector T cells systemically and in the tumor micro-environment (TME) [40•]. The scarcity of effector T cells in the tumor is compounded by enrichment of regulatory T cells (T_reg_), a subpopulation of CD4+ T cells that suppress effector T cells through cytotoxic T lymphocyte–associated protein 4 (CTLA-4) signaling and secretion of cytokines TGF-β and interleukin-10 [41, 42]. Additionally, the immune cell composition of the GBM TME is characterized by a large population of macrophage and myeloid-derived cells, as compared to the meager presence of lymphocytes [43]. Tumor-associated macrophages (TAMs) support the progression of GBM through promoting angiogenesis and suppressing the adaptive immune response [44]. Collectively, solid tumor cancers, principally GBMs, that bear enrichment of macrophages are associated with shorter survival compared to tumors without such enrichment [43]. Myeloid-derived suppressor cells (MDSCs) further dampen the adaptive immune response by suppressing the proliferation and functionality of tumor-infiltrating T cells through the production of anti-inflammatory cytokines and T cell suppressive compounds as well as upregulation of the transmembrane protein, programmed death-ligand 1 (PD-L1) [45–47]. In addition to these complex cellular mechanisms, GBM-associated hypoxia results in over-production of angiogenic factors including vascular endothelial growth factor (VEGF) and hypoxia-inducible factor1-alpha (HIF-1a), creating an irregular vascular network that results in limited access to nutrients, immune cells, and therapeutic treatments [48].

### Modulation of Centralized Signaling Pathways by Glioblastoma Promotes Immunosuppression

The ability of GBM to escape immune surveillance through deregulated signaling pathways enhances its ability to overcome many therapeutic strategies, including immunotherapies. For example, TGF-β, a major GBM-secreted factor, contributes to immunosuppression, angiogenesis, and maintenance of the glioma progenitor population through its involvement in multiple signaling pathways [49]. In addition, both GBM tumor cells and infiltrating immune cells induce constitutive activation of signal transducer and activator of transcription 3 (*STAT3*), which drives multiple pro-oncogenic pathways and dampens the anti-tumor immune response through recruitment and expansion of T_reg_ cells and MDSCs, induction of T cell tolerance through STAT3 hyper-activation in APCs, and regulation of immunosuppressive cytokines [50]. However, while effective in preclinical settings of a variety of different cancers including GBM, inhibition of STAT3 fails to provide a viable therapeutic option as STAT3 regulates many other necessary biological processes, and inhibition results in unintended side effects such as thrombocytopenia [51]. In addition, the fibrinogen-like protein 2 (*FGL2*) pathway promotes immunosuppression through increasing expression of programmed cell death-1 (PD-1) on T cells and by expanding populations of TAMs, MDSCs, and T_reg_ cells in the TME [52].

### Suppression of the Immune Response by Standard Therapy and Corticosteroids

Patients with high-grade glioma that undergo standard-of-care (SOC) treatment with concurrent radiation and alkylating temozolomide (TMZ) may demonstrate CD4+ lymphopenia, which increases the patient’s risk for infections including *Pneumocystis jiroveci pneumonia* (PJP, formally known as PCP) [53]. In addition, patients are also often treated with dexamethasone, a synthetic glucocorticoid, for peri-operative control of intracerebral edema and consequent neurological symptoms (e.g., headaches, confusion, weakness, other focal neurological deficits, and seizures). However, dexamethasone and other glucocorticoids significantly dampen the overall immune response and can reduce therapeutic benefit of immunotherapies, resulting in consensus recommendations that their use be minimized prior to and during immunotherapy trials [54–56]. For example, it was recently reported that use of dexamethasone hampered vaccine-reactive T cell responses during neoantigen vaccine priming in patients with newly diagnosed malignant glioma [56, 57].

## Recent Advances in Immunotherapy

**Fig. 2:**
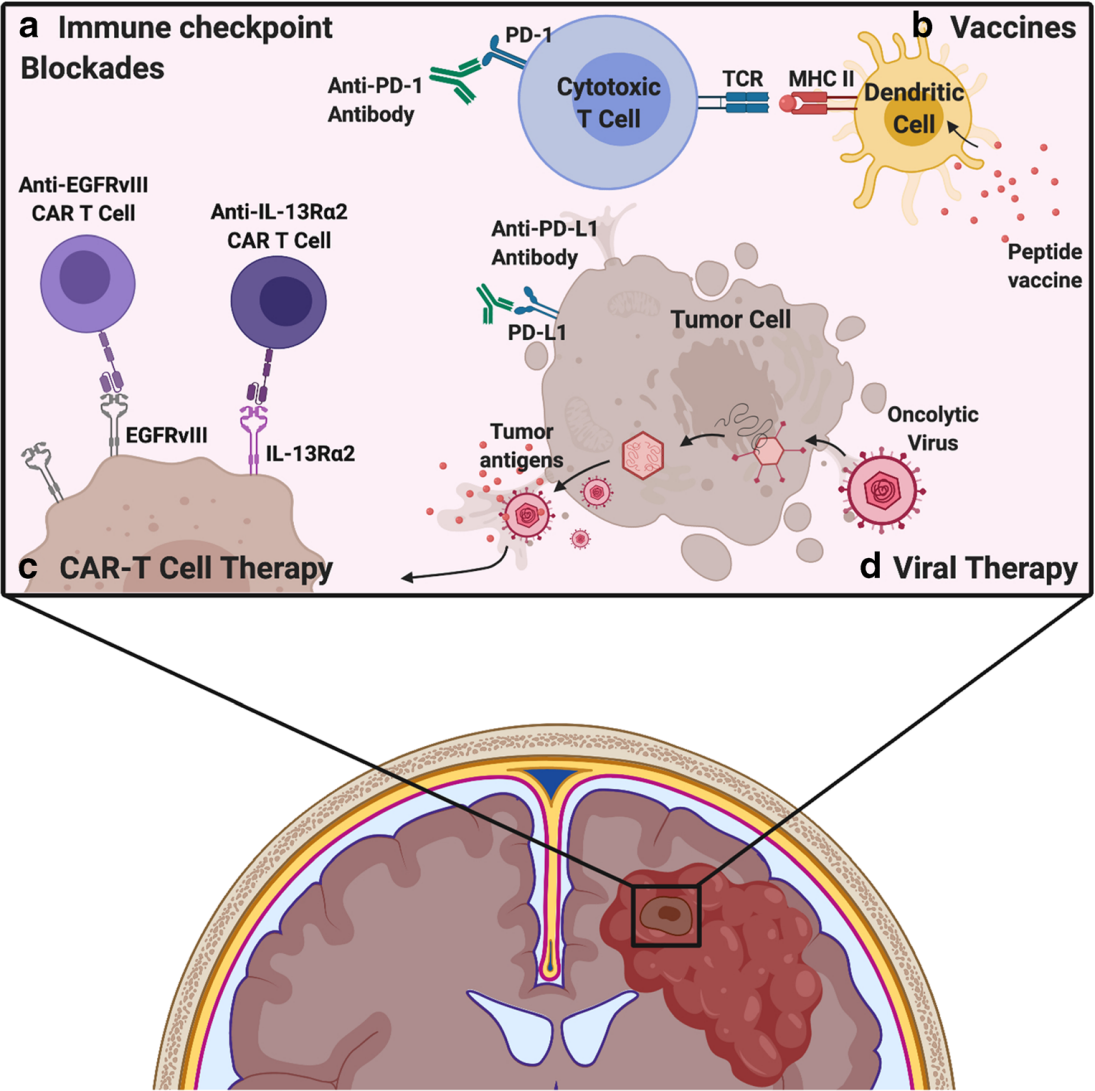
Immunotherapeutic strategies for treatment of glioblastoma. **a** Immune checkpoint receptor/ligands such as PD-1 expressed on T cells and PD-L1 expressed on tissue cells downregulate the adaptive immune response in normal tissues. Tumors may express PD-L1 as well, thus inhibiting T cell activation in tumors. Immune checkpoint inhibitors are antibodies that block receptor-ligand interactions, such as between PD-1 and PD-L1, thus inhibiting the immunosuppressive effects of this interaction. **b** Vaccines introduce GBM-specific antigens to native APCs including dendritic cells and rely on MHC-dependent presentation to T cells to stimulate a GBM-targeted immune response. **c** CAR-T cell therapy uses autologous T cells, which are genetically modified to target GBM-specific surface antigens, such as EGFRvIII and IL-13Rα2. Unlike vaccines, CAR-T cells do not rely on MHC-dependent antigen presentation. **d** Viral therapy encompasses the use of oncolytic viruses and retroviruses to either initiate tumor cell lysis and release of tumor antigen or to integrate therapeutic transgenes for expression by the tumor cell

Over the past few decades, a multitude of immunotherapeutic strategies have attempted to eradicate and overcome GBM, including CAR-T cell, vaccine, immune checkpoint inhibitors (ICI), and OV therapies, discussed in detail below and summarized in Fig. 2.

### Cell-Based Therapy: Chimeric Antigen Receptor T Cell Therapy

The purpose of cell-based therapy is to enhance the anti-tumor activity of patient-derived T cells by engineering them to express either a chimeric antigen receptor (CAR) or a T cell receptor (TCR) targeting specific tumor antigens. However, this section will focus primarily on CAR-T cell therapy [58]. Unlike CAR-T cells, TCR-modified T cells rely on major histocompatibility class (MHC) peptide presentation, which GBM can suppress, thereby evading MHC-restricted T cell recognition and rendering TCR-modified T cells incapable of recognizing tumor-associated antigens (TAAs) [59]. To circumvent this issue, CAR-T cells are engineered to express a CAR, composed of a single-chain variable fragment specific to the target, a T cell activation domain (CD3ζ), and one or more co-stimulatory domains (such as 4-1BB, CD28, OX40), which redirects T cell specificity to an extracellular TAA [59, 60].

In a recent phase I study, autologous CAR-transduced T cells targeting the constitutively active *EGFR* variant EGFRvIII, which is present in 24–67% of GBM cases, were intravenously administered to 10 recurrent GBM patients. The mOS was 8.25 months, and 4 patients who underwent re-resection within 14 days of treatment demonstrated infiltration of CAR-T cells and increased activated lymphocytes within the resected tumor [61]. However, another phase I trial, which utilized a third-generation CAR construct of EGFRvIII, did not show therapeutic benefit [62]. The glioma-associated antigen interleukin-13 receptor alpha 2 (IL13Rα2), found in approximately 58% of adult GBM cases and 83% of pediatric brain tumors, has also been targeted by CAR-T cells in the clinical setting [63, 64]. Preliminary results for an ongoing phase I trial with IL-13α2 CAR-T cells demonstrated radiographic response of both intracranial and metastatic spinal tumors in one patient for 7.5 months [63, 65]. Interestingly, initial intracavitary CAR-T cell delivery resulted in localized tumor control with no effect on distant tumor sites, but there was significant regression of disseminated disease after adjunctive intraventricular delivery [65]. Other CAR-T clinical trials targeting single antigens in GBM have been largely unsuccessful; however, novel targets are currently under clinical investigation (Table 1).

**Table 1:** Compilation of active or completed clinical trials investigating immunotherapeutic modalities in GBM, IDH mutant glioma, and pediatric DIPG/DMG diseases (2015–2020)

Trial name	Treatment	Phase	Diagnosis	Outcomes
GBM: VACCINES				
ReACT NCT01498328	1) Rindopepimut/GM-CSF 2) Bevacizumab + KLH	II (*n* = 73)	Recurrent EGFRvIII + GBM	1) PFS-6: 28% 2) PFS-6: 16% (*P* = 0.12, one-sided) [66]
ACT IV NCT01480479	1) Rindopepimut/GM-CSF + TMZ 2) KLH + TMZ	III (*n* = 745)	Newly diagnosed EGFRvIII + GBM	1) mOS: 20.1 mos. 2) mOS: 20.0 mos. (HR = 1.01, 95% CI 0.79–1.30, *P* = 0.93) [7]
IMA-950 NCT01920191	IMA950 multi-peptide vaccine + poly-ICLC	I/II (*n* = 16)	Newly diagnosed, HLA-A2 + GBM	mOS: 19 mos. (95% CI: 17.25–27.87) [67]
ICT-107 NCT01280552	1) ICT-107 (peptide-pulsed DC vaccine) 2) Un-pulsed DCs	II (*n* = 124)	Newly diagnosed GBM	1) mOS: 17.0 mos. 2) mOS: 15.0 mos. (HR = 0.87, *P* = 0.58) [68]
GAPVAC-101 NCT02149225	1) APVAC1 or APVAC2 (multi-peptide vaccines)/GM-CSF + poly-ICLC + TMZ	I (*n* = 16)	Newly diagnosed, HLA-A*02:01 or HLA-A*24:02 + GBM	mPFS: 14.2 mos. mOS: 29 mos. (for *n* = 15) (*P* = N/A) [69]
DCVax-L NCT00045968	1) DCVax-L (tumor lysate-pulsed DC vaccine) 2) PBMC (control)	III (*n* = 348)	Newly diagnosed GBM	mOS: 23.1 mos. (95% CI 21.2–25.4; *P* = N/A) [70]
NCT02078648	SL701/GM-CSF + poly-ICLC and bevacizumab	I/II (*n* = 74)	HLA-A2 + recurrent GBM	Stage 1 OS-12: 37% Stage 2 OS-12: 43% (*P* = N/A) [71]
I-ATTAC NCT03927222	CMV pp65-LAMP mRNA-pulsed DCs/GM-CSF/Td + TMZ	II (*n* = 48)	Newly diagnosed, CMV + MGMT unmethylated GBM	Recruiting
ACTION NCT03334305	TTRNA (tumor RNA) pulsed DCs/GM-CSF/Td ± HSCs	I (*n* = 8)	HGG	Recruiting
NCT01204684	Tumor lysate-pulsed DCs ± resiquimod or poly-ICLC	II (*n* = 60)	New or recurrent HGG	Active
NCT03382977	VBI-1901/GM-CSF	I/II (*n* = 38)	Recurrent, IDH-1/2 wildtype GBM	Recruiting
GBM: Checkpoint inhibitors				
CheckMate 143 NCT02017717	1) Nivolumab 2) Bevacizumab	III (*n* = 626)	Newly diagnosed, first recurrence, or MGMT unmethylated GBM	1) mOS: 9.8 mos. 2) mOS: 10.0 mos. (HR = 1.04; *P* = 0.76)[8•]
CheckMate 498 NCT02617589	1) Nivolumab + RT 2) TMZ + RT	III (*n* = 550)	Newly diagnosed, MGMT Unmethylated GBM	Failure to reach primary objective of OS
CheckMate 548 NCT02667587	1) Nivolumab + TMZ + RT 2) TMZ + RT	III (*n* = 693)	Newly diagnosed, MGMT Methylated GBM	Ongoing; failed to meet PFS primary endpoint; OS under investigation
NCT02337491	1) Pembrolizumab + bevacizumab 2) Pembrolizumab	II (*n* = 80)	Recurrent GBM	1) PFS-6: 26% mOS: 8.8 mos. 2) PFS-6: 6.7% mOS: 11.3 mos. [72]
NCT02313272	Pembrolizumab + bevacizumab + HFSRT	I (*n* = 32)	Recurrent HGG	OS-6: 94% OS-12: 64% [73]
NCT02794883	Durvalumab (anti-PD-L1) ± tremelimumab (anti-CTLA4)	II (*n* = 36)	Recurrent HGG	Active
NCT02968940	Avelumab (anti-PD-L1) + HFSRT	II (*n* = 43)	HGG	Completed
NCT02311920	Ipilimumab + nivolumab + TMZ	I (*n* = 32)	Newly diagnosed GBM or gliosarcoma	Active
GBM: Adoptive cell therapy				
NCT02208362	IL13Rα2-specific CAR T cells	I (*n* = 92)	Refractory/recurrent GBM	Ongoing; interval analysis PFS (*n* = 1): 7.5 mos. [65]
NCT02209376	EGFRvIII CAR T cells	I (*n* = 11)	Recurrent, EGFRvIII + GBM	mOS: ~ 8 mos. PFS: not evaluable [61]
NCT01454596	EGFRvIII CAR T cells, aldesleukin, fludarabine, and cyclophosphamide	I (*n* = 18)	New or recurrent, EGFRvIII + GBM	mOS: 6.9 mos. PFS: 1.3 mos. Outlier: 12.5 mos. [62]
HERT-GBM NCT01109095	HER.CAR CMV-specific CTLs	I (*n* = 16)	Recurrent, HER2 + CMV seropositive GBM	Ongoing; interim ORR (PR + SD): 33% [74]
NCT04077866	B7-H3 CAR-T + TMZ	I/II (*n* = 40)	Recurrent or refractory GBM	Recruiting
NCT04045847	CD147 CAR T cells	I (*n* = 31)	Recurrent CD147 + GBM	Recruiting
NCT04214392	Chlorotoxin CAR T cells	I (*n* = 36)	Recurrent MPP2 + GBM	Recruiting
GBM: Viral therapy				
DNX-2401 (Delta-24-RGD) NCT00805376	DNX-2401 (adenovirus) 1) IT injection 2) IT infusion, resection	I (*n* = 37)	Recurrent HGG	1) mOS: 9.5 mos. 2) mOS: 13.0 mos. (*P* = N/A) [75]
D24GBM NCT01956734	DNX-2401 + TMZ	I (*n* = 31)	Recurrent GBM	Ongoing; 3 objective responses (30, 19, and 27 mos.) [76]
PVSRIPO NCT01491893	1) Recombinant poliovirus 2) Historical controls	I (*n* = 61)	Recurrent GBM	1) mOS: 12.5 mos. (95% CI, 9.9 to 15.2) 2) mOS: 11.3 mos. (95% CI, 9.8 to 12.5) (*P* = N/A) [77]
NCT02986178	PVSRIPO + lomustine	II (*n* = 122)	Recurrent GBM	Active
NCT01156584	1) Toca 511 + Toca FC 2) External control	I (*n* = 54)	Recurrent, unresectable HGG	1) mOS: 13.6 mos. 2) mOS: 7.1 mos. (HR, 0.45; 95% CI, 0.27 to 0.77; *P* = 0.003) [78, 79]
NCT01470794	Toca 511 + Toca FC	I (*n* = 56)	Recurrent HGG	mOS: 11.9 mos. (95% CI, 10.7 to 15.1) [80]
NCT02414165	1) Toca 511 + Toca FC 2) SOC	II/III (*n* = 403)	Recurrent HGG; IDH-mut stratified	1) mOS: 11.1 mos. 2) mOS: 12.2 mos. (HR = 1.06; *P* = 0.6154) [6]
rQNestin NCT03152318	rQNestin34.5v.2 HSV-1 ± cyclophosphamide	I (*n* = 108)	Recurrent HGG	Ongoing
UMIN000002661	G47delta (2nd-gen. oncolytic HSV-1)	I-II (*n* = 21)	Recurrent GBM	Completed No results reported yet
UMIN000015995	1) G47delta 2) Historical control	II (*n* = 30)	Recurrent GBM	Ongoing 1) OS-12: 92.3% 2) OS-12: 15% [81]
NCT00390299	MV-CEA (measles virus) 1) IC injection 2) IT infusion + resection + IC injection	I (*n* = 23)	Recurrent GBM	1) mOS: 11.8 mos. PFS-6: 22.2% 2) mOS: 11.4 mos. PFS-6: 23.1% [82]
ParvOryx01 NCT01301430	H-1PV (H-1 parvovirus)	I/IIa (*n* = 18)	Progressive primary or recurrent GBM patients	mOS: ~ 15.5 mos. PFS: ~ 4 mos. (*P* = N/A) [83]
GBM: Combination therapies (not including SOC)				
Ad5-DNX-2401 NCT03896568	Ad5-DNX-2401 (adenovirus delivered in MSCs)	I (*n* = 36)	Recurrent, IDH-1 wildtype HGG/AAs	Ongoing
NCT03726515	EGFRvIII-targeted CAR-T + pembrolizumab	I (*n* = 7)	Newly diagnosed MGMT Unmethylated, EGFRvIII + GBM	Ongoing
NCT04003649	IL13Rα2 TN/MEM cells, ipilimumab and nivolumab (neoadjuvant, adjuvant)	I (*n* = 60)	Recurrent or refractory GBM	Recruiting
NeoVax NCT02287428	NeoAntigen (multi-peptide vaccine), RT, pembrolizumab	I (*n* = 46)	Newly diagnosed MGMT Unmethylated GBM	Ongoing
CAPTIVE/KEYNOTE-192 NCT02798406	DNX-2401, pembrolizumab	II (*n* = 49)	Recurrent GBM patients	Ongoing; interim analysis: OS-9: 100% (first 7 patients) [84]
NCT04013672	Pembrolizumab, SurVaxM (survivin vaccine)/GM-CSF, Montanide ISA 51	II (*n* = 51)	Recurrent GBM	Recruiting
IMA950-106 NCT03665545	Pembrolizumab + IMA950/poly-ICLC	I (*n* = 24)	Recurrent GBM	Ongoing
NCT03743662	Bevacizumab, nivolumab, RT, re-resection	II (*n* = 94)	Recurrent, IDH wildtype GBM	Recruiting
MEDI4736 NCT02336165	Durvalumab ± RT or bevacizumab	II (*n* = 159)	Newly diagnosed or recurrent MGMT Unmethylated GBM	Active
NCT03425292	Nivolumab ± ipilimumab ± bevacizumab ± TMZ	I (*n* = 90)	Newly diagnosed HGG	Recruiting
NCT04225039	SRS + GITR agonist (INCAGN01876) + anti-PD1 (INCMGA00012)	II (*n* = 32)	Recurrent, IDH wildtype GBM	Recruiting
NCT01811992	Ad-hCMV-tk and Ad-hCMV-Flt3L (adenoviral vectors)	I (*n* = 19)	Newly diagnosed GBM	Ongoing
NCT03576612	AdV-tk + valacyclovir + nivolumab + RT + TMZ	I (*n* = 36)	Newly diagnosed HGG	Recruiting
GLOBE NCT02511405	1) VB-111, bevacizumab 2) Bevacizumab	III (*n* = 256)	Recurrent GBM	1) mOS: 6.8 mos. 2) mOS: 7.9 mos. (HR, 1.20; 95% CI: 0.91–1.59; *P* = 0.19) [85]
DNX-2440 NCT03714334	DNX-2440 (OX40L adenoviral vector)	I (*n* = 24)	Recurrent GBM	Ongoing
M032-HSV-1 NCT02062827	M032-HSV-1 (2nd-gen. HSV) with IL-12	I (*n* = 36)	Recurrent GBM	Ongoing
IDH mutant glioma: Vaccine				
RESIST NCT02193347	IDH peptide vaccine (PEPIDH1M) + TMZ	I (*n* = 24)	Recurrent Grade II glioma, IDH mutant	Ongoing
NOA-16 NCT02454634	IDH1R132H peptide vaccine, montanide, imiquimod	I (*n* = 39)	Newly diagnosed AA and GBM; IDH1R132H mutant	80% specific T cell response; 87% with specific humoral response [86]
IDH mutant glioma: Combination therapy				
NCT03532295	INCMGA00012 (anti-PD1) + epacadostat + bevacizumab + RT	II (*n* = 55)	Recurrent GBM IDH wildtype or mutant	Recruiting
NCT03422094	NeoVax, ipilimumab, nivolumab	I (*n* = 3)	Newly diagnosed MGMT Unmethylated, IDH1/IDH2 mutant GBM	Ongoing
Pediatric: Adoptive cell therapy				
NCT04196413	GD2-CAR T cells	I (*n* = 54)	DMG with H3.3 K27M mutation	Recruiting
NCT04185038	B7H3-CAR T cells	I (*n* = 70)	DMG, or recurrent CNS tumor	Recruiting
NCT03389230	HER2(EQ)BBζ/CD19t+ T cells	I (*n* = 42)	Recurrent, HER2 + HGG	Recruiting
NCT03500991		I (*n* = 48)	Refractory or recurrent, HER2+ pediatric CNS tumor	Recruiting
NCT03638167	EGFR806-CAR T cells	I (*n* = 36)	EGFR + refractory or recurrent CNS tumor	Recruiting
Pediatric: Vaccine				
NCT02960230	H3.3 K27M peptide vaccine ± nivolumab	I (*n* = 29)	New glioma with H3.3 K27M mutation	Ongoing [56]
BRAVO NCT03396575	TTRNA (tumor RNA) pulsed DCs/GM-CSF/Td + HSCs + TMZ or cyclophosphamide/fludarabine	I (*n* = 21)	DMG	Recruiting
Pediatric: Immune checkpoint inhibition				
NCT04323046	Neoadjuvant ipilimumab ± nivolumab	I (*n* = 45)	Recurrent HGG	Not yet recruiting
Pediatric: Viral therapy				
NCT03178032	DNX-2401 + SOC	I (*n* = 12)	Naïve DIPG	Completed No results reported yet
NCT03043391	PVSRIPO	I (*n* = 12)	Recurrent HGG	Recruiting
NCT03911388	HSV-G207	I (*n* = 15)	Recurrent GBM and CNS tumors	Recruiting
Pediatric: Combination therapy				
NCT00634231	AdV-tk plus valacyclovir + RT	I (*n* = 12)	Malignant glioma or recurrent ependymoma	Active OS (*n* = 3): 24+ mos. PFS (*n* = 2): 37.3 and 47.7 mos. [87]

Clinical trial information was found on www.clinicaltrials.gov accessed August 5, 2020. IV intravenous, *mOS* median overall survival, *PFS-#* progression free survival-number of months, *OS-#* overall survival-number of months, *CI* confidence interval, *HR* hazard ratio, *ORR* overall response rate, *PR* partial response, *SD* stable disease, *SOC* standard of care, *TMZ* temozolomide, *KLH* keyhole limpet hemocyanin, *DC* dendritic cell, *IT* intratumoral, *IC* intracavitary, *RT* radiotherapy, *HFSRT* hypofractionated radiation therapy, *Td* tetanus diphtheria toxoid, *HSCs* hematopoietic stem cells, *MSCs* mesenchymal stem cells, *SRS* stereotactic radiosurgery, *DIPG* diffuse intrinsic pontine glioma

While hematologic cancers have been successfully treated with CAR-T cell therapy, its efficacy against CNS and other solid tumors has been limited by a host of barriers including heterogeneously expressed tumor antigens, limited persistence and homing to distant tumor sites, and TME-mediated immunosuppression [88]. To address GBM heterogeneity, there are several exploratory CAR-T systems in development that target multiple antigens, either in parallel or in tandem [89–93]. Additionally, improving CAR-T persistence within the CNS requires refinement of delivery, timing, and frequency and has been limited by incomplete understanding of CAR-T cell viability after CNS entry. In a phase I clinical trial with EGFRvIII CAR-T cells delivered through a single intravenous infusion, peak peripheral expansion of CAR-Ts was noted ~ 3–10 days after infusion, and detected up to 30 days after infusion, as defined by flow cytometry of peripheral blood samples [61]. Although this study looked at in situ tumor engraftment of CAR-T cells in tumors that were later resected, sample size was small (*n* = 7) and engraftment variable, so it is difficult to determine if tumor engraftment is related to amount and duration of CAR-T cells in the periphery or other factors [61]. Intratumoral, intracavitary, and/or intraventricular delivery may further improve durability of response. In comparison to intravenous therapy, preclinical studies have suggested that intraventricular (intrathecal) delivery may lead to faster tumor infiltration, increased penetration into the tumor core, and longer persistence within the tumor [94, 95]. However, preliminary evidence in one patient who received intrathecally delivered CAR-Ts targeting IL13Rα2 showed that CAR-T cells may persist in the cerebrospinal fluid for only 7 days, although this study did not look at CAR-T infiltration into the tumor itself [65]. Delivery issues including method and frequency need further investigation and would benefit from improved, non-invasive monitoring techniques, as discussed later.

### Immune Checkpoint Inhibitors

Despite success in other cancers, ICI blockade of PD-1/PD-L1 and CTLA-4 has been grossly unsuccessful thus far in GBM. A phase III trial comparing the PD-1 inhibitor nivolumab to VEGF-A inhibitor bevacizumab showed no improvement in survival in patients with recurrent GBM (CheckMate-143) [8•]. The Checkmate-498 trial, comparing nivolumab plus radiation (RT) versus standard-of-care temozolomide plus RT in patients with newly diagnosed GBM without *MGMT* promoter methylation (a poor prognostic factor), was stopped after nivolumab with radiation did not show improved survival compared to temozolomide with radiation (NCT02617589, publication pending). There is a complementary ongoing trial in patients with newly diagnosed *MGMT* promoter methylated GBM (CheckMate-548, NCT02667587). Preliminary results have shown that there is no improvement in progression-free survival (PFS) with the addition of nivolumab to temozolomide and RT, but final results regarding OS are pending. Several ongoing trials are also exploring the combination of nivolumab with the CTLA-4 inhibitor, ipilimumab (Table 1).

Interestingly, while checkpoint inhibition has been mostly ineffective in GBM, there has been some efficacy demonstrated in brain metastases (BMs) from systemic cancers including melanoma and non-small cell lung cancer [96–98]. The discrepancy in efficacy of ICI therapy between BMs and GBM may be partially explained by the variation in immune cell phenotype, function, and spatial organization of each tumor type’s respective TME [99]. Two recent, comprehensive analyses of the TME in both primary and metastatic brain malignancies found that BMs, especially melanomas, were characterized by higher proportions of lymphocytes, including CD8+ T cells, unlike gliomas, which instead exhibited an abundance of tissue-resident microglia and myeloid cells [99, 100••]. The efficacy of ICIs in metastases may also relate to neuroanatomical differences; metastases have BBB remodeling that may allow for more efficient immune cell intravasation [101, 102]. In addition, metastases are often well circumscribed, and a higher percent volume of each metastasis has BBB disruption, compared to diffusely infiltrating astrocytomas that have large areas of tumor with an intact BBB [103].

Other factors contributing to the failure of ICIs in GBM may include decreased passage through the BBB of ICI and activated T cells, as well as low expression of neoantigens by GBM compared to other systemic tumors [104]. As there are few tumor-infiltrating lymphocytes (TILs) in GBM [105], maximal efficacy of checkpoint inhibitors may require initial peripheral activation of circulating T cells [106•]. There is an ongoing study to evaluate if there is utility in combining intravenous/intrathecal delivery of PD-1 inhibitors in brain metastases of melanoma (NCT03025256), but no such trial has yet been undertaken in primary brain tumors. Compounding the lack of access of CTLA4-expressing APCs and PD-1-expressing T cells to the CNS, not all GBMs express PD-L1, and those that do show PD-L1 expression in fewer than 5% of cells [107–110]. In addition, the tumor mutational burden and neoantigen expression are lower compared to systemic cancers [107–110]. Furthermore, patients with GBM often have impaired systemic immunity, limiting the potential response to checkpoint inhibition [40•]. With ICI exposure, there may be compensatory upregulation of the tumor-driven immunosuppressive pathways, including upregulation of other checkpoint pathway ligands that could cause T cell exhaustion. Finally, there may be some relevance to ICI timing, as a recent small trial demonstrated improved survival in GBM with treatment with the anti-PD-1 antibody, pembrolizumab, prior to surgery, suggesting possible utility to immune system priming prior to antigen exposure during surgery [111•].

### Vaccine Therapy

Given the immunologically “cold” environment of GBM, efforts have been made to enhance the adaptive arm of the immune system to target GBM cells through cell-mediated immunity [112]. Three peptide or dendritic cell (DC)-based vaccines recently reached phase III clinical trial testing. In the phase III trial Act IV, rindopepimut, an immunogenic peptide vaccine targeting EGFRvIII, elicited a strong humoral response but conferred no significant improvement in PFS or mOS [7]. Durability of response may have been hampered by acquired loss of EGFRvIII expression, illustrating one of the greatest shortcomings of single-antigen vaccines [7]. On the other hand, a multi-peptide DC vaccine, ICT-107, targeting six GBM-specific HLA-A1 and A2 antigens was tested in a randomized phase II trial and prolonged patient PFS to 11.4 months, a significant increase compared to the control group’s PFS of 10.1 months (HR = 0.64, *p* = 0.033) [68]. ICT-107 was most effective in extending the mOS and PFS in HLA-A2+ patients or patients with *MGMT*-methylated GBM [68]. A follow-up phase III trial testing ICT-107 was attempted, but was discontinued due to inadequate funding. The third vaccine to reach phase III testing is DCVax-L, derived by educating autologous DCs with whole tumor lysate extracted from individual patient resections. The interim results of the placebo-controlled phase III DCVax-L trial showed an mOS of 23.1 months; however, the analysis included all patients and was not broken down by treatment group [69, 70].

Other single- and multi-antigen vaccines are under investigation in early phase trials, including (IDH1) R132H and H3.3K27M peptide vaccines. The (IDH1) R132H peptide vaccine targets a frequently occurring mutation in the IDH1 gene found primarily in lower grade gliomas, which is responsible for aberrant neural signaling and glioma progression [35]. In the phase I NOA-16 trial, this vaccine was administered with SOC in newly diagnosed patients with (IDH1) R132H-positive WHO Grade III and IV astrocytomas. Preliminary results demonstrated induction of a humoral immune response with a reasonable toxicity profile [86]. In a recent multicenter pilot study, vaccine targeting H3.3K27M, along with Toll-like receptor 3 agonist, polyinosinic-polycytidylic acid stabilized with polylysine and carboxy-methylcellulose (poly-ICLC), was administered to HLA-A02.201+ pediatric patients with H3.3K27M+ diffuse midline glioma (DMG). A subset of patients (*n* = 18) were selected for available multi-timepoint blood draws and 39% exhibited an expansion of H3.3K27M-reactive CD8^+^ T cells (mOS 16.3 months) and showed significantly prolonged OS compared to tumors who failed to demonstrate a T cell response (mOS 9.9 months) [56]. Furthermore, dexamethasone administration is inversely associated with H3.3K27M-reactive CD8^+^ T cell responses [56].

Multi-peptide vaccines are designed to elicit a strong T cell response to multiple tumor antigens. For example, IMA-950 (multi-peptide vaccine composed of 9 MHC class I and 2 MHC class II peptides), with poly-ICLC, was recently tested in a phase I clinical trial and resulted in patients developing tumor peptide-specific CD4+ and CD8+ T cell responses, with the IMA-950 antigens remaining stably expressed on the tumor throughout the course of the disease [67]. Another multi-peptide vaccine was tested in the GAPVAC-101 trial, in which personalized cocktails of pre-manufactured peptide vaccines utilized non-mutated GBM antigens (APVAC1) and neoepitopes (APVAC2) to elicit an immunological response. This trial demonstrated sustained memory CD8+ T cell responses induced by APVAC1, while a T_H_1 CD4+ T cell response was elicited by APVAC2 [113]. Another recent phase I/Ib study tested the efficacy of a personalized neoantigen vaccine in newly diagnosed GBM patients and found the vaccination promoted neoantigen-specific CD4^+^ and CD8^+^ T cell responses and additionally increased the number of tumor-infiltrating T cells [57].

### Viral Therapy

Aberrant cellular pathways in GBM provide a favorable environment for specially modified viruses to selectively replicate [114]. Following successful replication within target tumor cells, oncolytic viruses (OVs) elicit lytic cell death, provoking a strong inflammatory response followed by a tumor-targeted immune response, initiated by the liberation of damage-associated molecular patterns (DAMPs) and TAAs from tumor cells [115, 116]. A variety of viruses have been reengineered to target GBM and have been critically reviewed by *Chiocca* et al. (2019) [117••]*.*

Adenoviruses can be readily manipulated to drive cell death, facilitate an immunogenic anti-tumor response, or carry therapeutic transgenes [118]. For example, conditionally replicative adenoviruses have been modified to selectively replicate in tumor cells with aberrant p16/Rb/E2F signaling (which controls the activity of the retinoblastoma tumor suppressor protein (Rb), a regulator of the E2F transcription factor) [119]. DNX-2401 (previously delta-24-RGD) selectively replicates within brain tumor stem cells with an abnormal p16INK4/Rb pathway [120]. Recently, a phase I clinical trial demonstrated that replication of DNX-2401 in recurrent GBM tumors facilitated tumor cell death and some immunogenic cell death by infiltrating CD8+ T cells and T_H_ cells, lengthening mOS to 13.0 months [75]. Another phase I trial in newly diagnosed GBM seeks to capitalize on putative synergy between DNX-2401 and temozolomide, improving tumor recognition by CD8+ T cells [121, 122]. Interim results reported several objective radiological responses up to 30 months [76]. G47delta, a conditionally replicating, double mutant derivative of herpes simplex virus-1 (HSV-1), is another oncolytic virus undergoing current investigation. In a phase II trial in recurrent/residual glioblastoma, G47delta was injected intracranially in combination with adjuvant TMZ, resulting in an influx of lymphocytes, and interim results have reported a 1-year survival of 92.3% [123].

Unlike OVs, replicating retroviruses (RRVs) are non-lytic, require an actively dividing host cell to support viral replication, and can act as selective carriers of exogenous pro-cytotoxic transgenes [124]. Toca 511 (vocimagene amiretrorepvec) is an RRV carrying a modified yeast-derived cytosine deaminase gene (CD) that integrates into the tumor cell genome and converts systemically delivered pro-drug, 5-fluorocytosine (5-FC) into cytotoxic 5-fluorouracil (5-FU) [124]. Despite promising phase I results, the phase II/III trial failed to improve OS as compared to a SOC cohort, with mOS of 11.1 and 12.2 months, respectively, leading to premature termination [6]. It should be noted that certain factors predicted improved survival, namely diagnosis of anaplastic astrocytoma, presence of IDH1 R132H mutation, and second disease recurrence.

## Moving Forward

Thus far, immunotherapy has had minimal impact on the survival of GBM patients, challenged by limitations with antigen presentation, immune cell trafficking into the CNS, tumor infiltration, and immunosuppression from the tumor and its treatments. Additionally, accurate assessment of the efficacy of immunological therapies is challenged by the limited functionality of non-invasive monitoring tools, resulting in cases of premature treatment discontinuation and a limited understanding of treatment impact.

### Overcoming Adaptive Immune Resistance and Immune Escape

Novel therapies need to address mechanisms of immune escape, including T cell exhaustion and adaptive resistance [125]. One strategy addressing T cell exhaustion includes co-administration of a glycogen synthase kinase 3 (GSK3) inhibitor with CAR-T cell therapy as there is some evidence that this may reduce CAR-T exhaustion and help establish an effector memory T cell population [126]. Due to some evidence that the PD-L1 ligand may be upregulated after CAR-T therapy [61, 127], one ongoing trial has incorporated checkpoint inhibition in conjunction with CAR-T (Table 1). Another similar strategy involved modification of Delta-2401 (previously delta-24-RGD) adenovirus to deliver the gene for the OX40 ligand T cell stimulatory gene to glioma tumor cells. An exploratory study administered this modified virus (Delta-24-RGDOX or DNX2440) with a PD-L1 inhibitor and noted an increase in infiltrative T cells in vivo in a preclinical tumor model, leading to the initiation of a phase I clinical trial (see Table 1) [128]. Finally, anti-CTLA-4 (ipilimumab) and anti-PD1 (pembrolizumab) have been used in clinical trials and are currently undergoing investigation in combination with a vaccine therapy (Table 1).

Advances in gene therapy offer a multimodal approach to targeting GBM. In an ongoing phase I trial, patients were given intratumoral injections of an adenovirus encoding HSV type I thymidine kinase (Ad-TK), which converts systemically delivered valacyclovir to cytotoxic ganciclovir within infected tumor cells, in addition to a second adenovirus delivering the dendritic cell (DC)–recruiting Fms-like tyrosine kinase 3 ligand (Ad-Flt3L) to transform the tumors into DC-attracting hot zones. In mouse xenografts, this gene therapy was combined with a combinatorial PD-1/CTLA-4 blockade, which reduced the MDSC population and enhanced survival [129•].

Other combinatorial approaches have attempted to simultaneously target the lifeblood of the tumor while forcing tumor cells into apoptosis by using VEGF inhibitors and gene therapy. For example, in the phase III randomized GLOBE trial, VEGF inhibitor bevacizumab was combined with the VB-111 adenovirus, which delivers a transgene activating the Fas pro-apoptotic pathway in tumor endothelial cells; however, this combination did not extend mOS compared to bevacizumab monotherapy (Table 1) [85]. Of note, early phase trials with VB-111 had a median OS of 13.6 months, but VB-111 was administered concurrently with bevacizumab, rather then prior to bevacizumab as was the case in later trials [85]. Other ongoing combinatorial therapies are summarized in Table 1.

### Current Diagnostic Shortcomings and Solutions

Advancement in the use of immunotherapy in GBM will also require improved methods to assess immune response in the tumor. Unfortunately, this assessment currently relies on invasive biopsy, with limited functionality of non-invasive monitoring tools.

Magnetic resonance imaging (MRI) is limited in its ability to distinguish between tumor, treatment effect, and inflammatory infiltration [130]. The Immune Response Assessment in Neuro-Oncology (iRANO) guidelines for interpreting MRIs while on immunotherapy were released in 2015 [54]. These recommend deferring final interpretation of MRI changes for 3 months, a relatively long period for a disease with mOS of 20–22 months from initial diagnosis and approximately 9 months from recurrence. Other advanced imaging techniques, including MRI with delayed or dynamic susceptibility contrast, dynamic MRI perfusion, arterial spin labeling, diffusor tensor imaging, MR spectroscopy, and position emission tomography (PET), all lack the sensitivity, specificity, and/or resolution to reliably predict which areas of imaging correlate to tumor progression versus immune infiltrate [130]. There has been some effort to label lymphocytes ex vivo with a radionuclide or MRI probe at the cell surface, or to engineer cytolytic CD8+ T cells to express a radionuclide, PET reporter gene, or MRI reporter gene [131]. In other neuro-inflammatory conditions including multiple sclerosis (MS) and cerebral infarct, radioisomer C11-PK11195-labeled translocator protein can identify activated astrocytes, microglia, astrocytes, and macrophages imaged using PET [130, 132]. In MS, ultra-small paramagnetic iron oxide nanoparticles have also been used to image infiltrative immune cells where the BBB is intact [133]. These technologies could potentially be extended to immunotherapy in GBM.

## Conclusion

Therapeutic success with immunotherapy in GBM has been challenged by immune surveillance evasion through multiple deregulated signaling pathways, limited antigen presentation, and production of immunosuppressive cytokines and regulatory immune cells. These failures have emphasized that successful treatment of GBM may rely on combination therapies, with cell-based therapies targeting multiple specific antigens and therapies that indiscriminately target a broad population of GBM cells, such as oncolytic viruses. Furthermore, thoughtful design of dosing schedules and delivery methods of cell-based therapies, oncolytic viruses, immune checkpoint inhibitors, and vaccines is essential in affirming their best possible performance when used in the combination therapy setting.
